# A same‐day assay predicts apoptotic response to combined BCL‐2 and MCL‐1 BH3‐mimetic targeting in multiple myeloma cells

**DOI:** 10.1002/jha2.133

**Published:** 2020-11-20

**Authors:** Martin Grundy, Firas Al‐Kaisi, Joanna Cull, Cathy Williams, Dean Smith, Claire H. Seedhouse

**Affiliations:** ^1^ Blood Cancer and Stem Cells, Division of Cancer and Stem Cells School of Medicine University of Nottingham Biodiscovery Institute Nottingham UK; ^2^ Clinical Haematology Nottingham University Hospitals Nottingham UK

**Keywords:** myeloma, predictive assay, S63845, venetoclax

## Abstract

Recent advances in treatment options for multiple myeloma (MM) have positive impact on patient survival. However, there is a short fall of rapid and reliable assays that can predict patient response to novel agents. The anti‐apoptotic proteins B‐cell lymphoma‐2 (BCL‐2) and myeloid cell leukaemia‐1 (MCL‐1), are necessary for MM survival, although most myelomas are more dependent on MCL‐1. BCL‐2 inhibition alone yields significant cytotoxicity in only a minority of cases, therefore targeting both proteins simultaneously, is a therapeutic option. Venetoclax and S63845 are BCL‐2 and MCL‐1 targeting BH3‐mimetics which have demonstrated apoptotic synergy in MM. We investigated whether a novel short‐term flow cytometric cytochrome c release assay could predict response to dual BH3‐mimetic targeting in MM cells. Six human myeloma cell lines (HMCL) and seven primary samples were treated with venetoclax and S63845 alone or in combination. The 4‐hour assay confirmed the drug combination was synergistic in all HMCL tested. Annexin‐V data at 48 hours corresponded with 4‐hour response verifying the assay as a predictor of drug sensitivity. All primary samples responded to the drug combination, including samples with 1q gain and t(4;14) translocation. Normal stem cells were unaffected by the drug combination. We have developed a novel assay with the potential to predict response to therapy in MM cells.

## 1 INTRODUCTION

Multiple myeloma (MM) is the second most common haematological malignancy and is characterised by molecular complexity and clonal proliferation of plasma cells in the bone marrow [1]. Despite the use of high‐dose chemotherapy and autologous stem cell transplantation, the disease generally remains incurable, with treatment aiming to slow progression, alleviate symptoms and improve quality of life. The past decade has, however, seen significant advances in treatment options. The approval of novel agents, including proteasome inhibitors, immunomodulatory drugs, monoclonal antibodies and the developing CAR‐T‐cell therapy have positive impact on patient progression‐free survival and overall survival [2, 3]. However, MM is a heterogeneous condition and response to treatments is variable. There is, therefore, a requirement for sensitive approaches to compare the efficacy of therapies and real‐time assays to assess a patient's response to a drug or drug combination would be greatly beneficial.

A signature of MM is cell cycle dysregulation characterised by over‐expression of cyclin D proteins along with reliance on anti‐apoptotic members of the BCL‐2 (B‐cell lymphoma‐2) family for survival [4]. The emergence of BCL‐2 homology domain 3 (BH3) mimetics designed to target the anti‐apoptotic members of the BCL‐2 family has provided another therapeutic option. The apoptotic fate of a cell depends on a balance of interactions between pro‐survival molecules such as BCL‐2, MCL‐1 (Myeloid Cell Leukaemia‐1) and BCL‐_XL_ (B cell lymphoma‐extra‐large) and BH3‐only protein sensitiser molecules such as BAD (BCL‐2 associated death promotor) and NOXA (PMAIP1; Phorbol‐12myristate‐13‐acetate‐induced protein 1) [5]. Following a cell death stimulus, BH3‐only sensitiser proteins are activated, and displace BH3‐only activator proteins such as BIM (BCL‐2 interacting mediator of cell death) and BID (BH3 interacting‐domain death agonist) from their pro‐survival molecular chaperones, resulting in activation of effector molecules BAX (BCL‐2 associated X protein) and BAK (BCL‐2 homologous antagonist killer). BAX and BAK subsequently oligomerise and form pores that cause mitochondrial outer membrane permeabilisation and loss of mitochondrial outer membrane potential. This results in cytochrome c release from its usual location within the intermembrane mitochondrial space into the cytoplasm, leading to activation of the caspase cascade, and apoptosis.

Although both BCL‐2 and MCL‐1 are necessary for MM survival, most myelomas are dependent on MCL‐1 such that BCL‐2 inhibition alone only yields significant response in a minority of cases [6]. A dual targeting approach may therefore be beneficial. The most promising BCL‐2 inhibitor to date is the BH3‐mimetic venetoclax, which has demonstrated efficacy as targeted therapy for t(11;14) in relapsed/refractory myeloma [7]. Resistance to venetoclax monotherapy is associated with released BIM being sequestered by MCL‐1, and MCL‐1 is a known crucial pro‐survival factor in MM. Until recently a truly selective and potent small molecule inhibitor of MCL‐1 had yet to be developed, and a worrying caveat is that genetic deletion of MCL‐1 in mouse models resulted in bone marrow failure and myocardial toxicity [8]. It is hoped that transient inhibition of MCL‐1 using a BH3‐mimetic could circumvent these issues. S63845 is a novel MCL‐1 targeting BH3‐mimetic, that has shown low toxicity in pre‐clinical models and has demonstrated synergy in combination with venetoclax in haematological malignancies [9, 10, 11, 12]. We have previously reported that inhibiting MCL‐1 expression with S63845 produces a synergistic apoptotic response when used in combination with venetoclax in acute myeloid leukaemia (AML) cells [13].

In this era of novel medicines and targeted molecular therapies there is a short fall of predictive assays to demonstrate which drugs an individual patient will best respond to. This is particularly true in MM, where primary samples survive poorly in vitro away from the bone marrow niche, confounding conventional cytotoxicity assays. We have, therefore, focused on a same‐day flow cytometric cytochrome c release assay. We show that co‐operative targeting of BCL‐2 and MCL‐1 with venetoclax and S63845, induces a synergistic apoptotic response, in human myeloma cell lines (HMCL) and primary samples. Using the assay, we were able to record a short‐term (4‐hour) readout of response that was able to predict longer term (48‐hour) sensitivity to the drug combination. This assay could offer clinicians a faster and more targeted therapeutic window when compared to mouse patient‐derived xenograft models and colony assays.

## 2 MATERIALS AND METHODS

Drugs and reagent suppliers used in the study were as follows: venetoclax was supplied by Bioquote Limited (York, UK) and S63845 Active Biochem (Kowloon, Hong Kong). All other reagents were from Sigma (Poole, Dorset, UK) unless specified.

### 2.1 Primary samples

Bone marrow samples from MM patients or cells from G‐CSF mobilised donor normal stem cell harvests (NSCH) were obtained with written, informed consent according to the protocol approved by National Research Ethics Service Committee (NREC) East Midlands, Nottingham 1 or NREC East Midlands, Nottingham 2 and Nottingham University Hospitals NHS Trust. Mononuclear cells were purified from bone marrow via density‐gradient centrifugation method using Histopaque‐1077 according to the manufacturer's instructions. Plasma cells were isolated using CD138 microbeads (Miltenyi Biotec, Germany). Patient sample demographics are listed in Table 1.

**TABLE 1:** MM cell line characteristics

Cell line	IGH Translocation	P53	Other
MOLP‐8	t(11;14) (Cyclin D1)	WT	Near diploid, 1q amplification, 1p gain
KMS‐12‐BM	t(11;14) (Cyclin D1)	Mutated	hypertriploid, 1q (3‐4 copies)
U‐266	t(11;14) (Cyclin D1)	Loss	RB‐1null, hypodiploid, 1q gain, 1p gain
	Cyclin D2 expression		
OPM‐2	t(4;14) (Cyclin D2)	Mutated	1q amplification, c‐myc (8;14), hypertriploid, 1p gain
MM.1S	t(14;16) (Cyclin D2)	WT	1q gain
JJN3	t(14;16) (Cyclin D2)	No expression	C‐myc (8;14), hypotriploid, 1q amplification, 1p gain

### 2.2 Cell lines

MM.1S cells were obtained from the ATCC (Manassas, VA), JJN‐3, OPM‐2 and U‐266 from DSMZ (Braunschweig, Germany), and were maintained in RPMI‐1640 medium with 10% foetal calf serum (FCS) and 2 mM L‐glutamine. KMS.12.BM and MOLP‐8 HMCL were obtained from DSMZ and maintained as above with 20% FCS. Cultures were sustained at 37°C, 5% CO_2,_ and experiments were performed with HMCL in log phase. Testing to authenticate cells was performed using multiplex short tandem repeat analysis (Promega, UK). Mycoplasma testing was performed using the Mycoalert mycoplasma detection kit.

### 2.3 Cytochrome c release assay

Dose‐response assays were performed to select a drug concentration that produced 10‐20% cytochrome c release as a single agent. Note that 10 μM venetoclax was determined for all HMCL except KMS.12.BM where 1μM was used. Concentrations of 0.005μM (MOLP‐8), 0.01μM (KMS.12.BM and OPM‐2), 0.1μM (JJN‐3), 0.5μM (U‐266) and 10μM (MM.1S) were determined for S63845. HMCL were incubated at 5 × 10^5^/ml in culture medium for 4 hours with venetoclax, S63845 or the drug combination. Primary MM samples were incubated in culture medium for 4 hours with 1μM venetoclax, 1μM S63845 or the combination. Cells were fixed in 2% para‐formal dehyde, permeabilised with saponin and labelled with Alexa‐647‐cytochrome c antibody to determine the percentage of cells with loss of cytochrome c. Plasma cells were identified using a CD138 PE, CD19 APC‐H7 and CD38 FITC antibody panel. Data were collected on a FACSCanto II flow cytometer and analysed with FACS Diva software. All antibodies were obtained from Becton Dickinson, UK.

### 2.4 Annexin‐V analysis

Dose‐response assays were performed to select a drug concentration that produced 10‐20% apoptosis as a single agent. Note that 10 μM venetoclax was determined for all HMCL except KMS.12.BM where 1μM was used. Also 0.005 μM (MOLP‐8), 0.01μM (KMS.12.BM), 0.1μM (JJN‐3), 0.5μM (U‐266) and 10μM (MM.1S and OPM‐2) were determined for S63845. HMCL were incubated at 2.5 × 10^5^/mL for 48 hours with venetoclax, S63845 or the drug combination. The percentage of apoptotic cells after 48 hours was determined using a TACS Annexin‐V‐FITC Apoptosis Detection Kit (R&D Systems, UK).

### 2.5 Western blot analysis

HMCL were treated for 4 hours with the previously stated drug combinations. Cell lysates were prepared, separated by SDS‐PAGE, and transferred to nitrocellulose membranes. Detection antibodies included anti‐MCL1 and anti‐Bcl‐2 from Santa Cruz Biotechnology (Santa Cruz, CA) anti‐BCL‐_XL_ (Bethyl laboratories, TX), anti‐Cyclin D1, anti‐Cyclin D2 and anti‐β‐actin were from Abcam (Cambridge, UK).

### 2.6 Calculations and statistics

Fold excess additivism was calculated as a ratio of observed to expected values for drug combinations, where the expected value C is calculated from the Bliss algorithm for response to two compounds with effects A and B that is C = A + B − A × B [14]. Statistical analysis was performed using the Statistical Package for Social Sciences, version 23. *P* values of ≤.05 were considered to represent significance.

## 3 RESULTS

### 3.1 A short‐term cytochrome c release assay predicts apoptotic response to BH3‐mimetic combination therapy in MM cell lines

**FIGURE 1:**
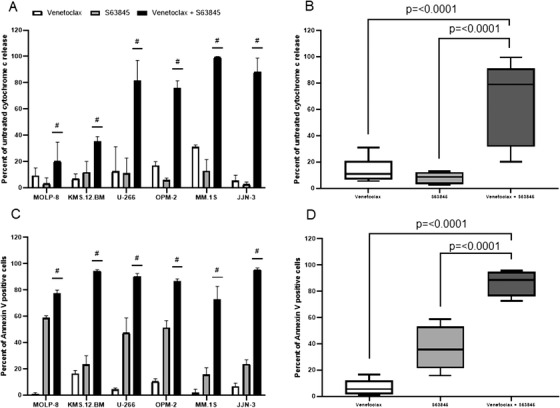
A short‐term cytochrome c release assay predicts long‐term apoptotic response to the combination of venetoclax and S63845. A, HMCL were treated with venetoclax, S63845 as single agents or in combination. Following 4‐hour drug treatment, cells were fixed and processed for cytochrome c release. Hashtags indicate synergy according to the bliss independence model as described in the methods. Columns, mean of three independent experiments; bars, SD. B, Summary of the median interquartile range (boxes) and range (error bars) of cytochrome c release in 6 HMCL treated with venetoclax, S63845 as single agents or in combination (*P = <.0001). C, HMCL were treated with venetoclax, S63845 alone or in combination. After 48 hours, cells were assayed for Annexin‐V positivity. Hashtags indicate synergy according to the bliss independence model as described in the methods. Columns, mean of three independent experiments; bars, SD. D, Summary of the median interquartile range (boxes) and range (error bars) of Annexin‐V positivity in 6 HMCL treated with venetoclax, S63845 alone or in combination (*P = <.0001)

HMCL were treated for 4 hours with venetoclax and S63845 alone or in combination followed by measurement of cytochrome c release. Dose‐response assays were performed to select a drug concentration that produced minimal (10‐20%) cytochrome c release as a single agent (data not shown). It has previously been reported that venetoclax monotherapy is effective in some, but not all, MM patients with t(11;14), and these patients have relatively high BCL‐2 gene expression compared with MCL‐1 and BCL‐_XL_ [15]. All six HMCL were relatively resistant to venetoclax monotherapy when compared to the sensitivity seen with S63845. It was noted however that the most sensitive cell line to single agent venetoclax was KMS.12.BM, which has t(11;14), thus supporting the validity of our assay (IC_10‐20_ 1μM KMS.12.BM compared to the other five HMCL where IC_10‐20_ 10μM). A strong synergistic apoptotic response was observed with the combination of venetoclax and S63845 in all the HMCL tested (Figure 1A and B). Translocations leading to cyclin D2 upregulation including t(4;14), t(14;16) and t(14;20) are associated with a poor prognosis in myeloma patients [16]. Of note, drug synergy was particularly pronounced in all four HMCL (U‐266, OPM‐2, MM.1S and JJN3) with upregulated cyclin D2 (Table 2). No correlation was observed with cell line p53 status and drug response.

**TABLE 2:** Primary sample demographics

Sample ID	Gender	Age (years)	Cytogenetics
MM2	Female	69	t(4;14) FGFR3‐IGH rearrangement
MM4	Male	85	Normal
MM5	Female	71	1q gain
MM18	Male	75	ATM gain
MM19	Male	41	Normal
MM22	Male	67	1p and 1q gain, TP53 gain, IGH gain
MM23	Male	52	1q gain

We have previously reported that 4‐hour BH3 peptide‐driven cytochrome c release can predict long‐term response to chemotherapeutic drugs [17]. We investigated whether 4‐hour drug induced cytochrome c release could predict longer term response in MM cells. HMCL were treated for 48 hours with venetoclax and S63845 alone or in combination and then subjected to Annexin‐V analysis to determine percentage of apoptotic cells. Again, dose‐response assays were performed to select a drug concentration that produced minimal apoptosis as a single agent (data not shown). Apoptotic response to the drug combination after 48 hours closely mimicked 4‐hour apoptotic response measured using cytochrome c release thus confirming that the cytochrome c assay has the potential to predict longer term drug response (Figure 1C and D).

### 3.2 Modulation of pro‐survival BCL‐2 family members and cyclin D proteins following venetoclax and S63845 treatment

**FIGURE 2:**
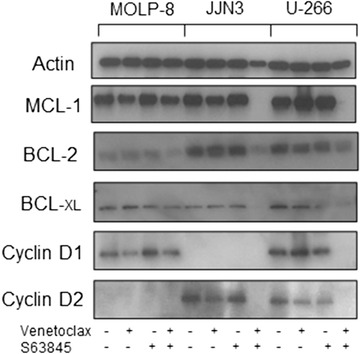
The combination of venetoclax and S63845 causes decreased expression of pro‐survival BCL‐2 family members and cyclin D proteins. MCL‐1, BCL‐2, BCL‐_XL_, Cyclin D1 and Cyclin D2 protein expression in untreated HMCL or cells treated for 4 hours with venetoclax, S63845 or the drug combination. The blots shown are cropped and are an example of three independent experiments

We have previously reported apoptotic synergy with venetoclax using various drugs that target MCL‐1 non‐specifically. All of these agents caused MCL‐1 protein degradation as single agents after 4 hours [18]. Here we demonstrate no reduction in MCL‐1 protein expression following 4‐hour S63845 single agent treatment, highlighting its different mechanism of action on MCL‐1 inhibition (Figure 2).

### 3.3 A short‐term cytochrome c release assay can measure apoptotic response to BH3‐mimetic combination therapy in primary MM cells

**FIGURE 3:**
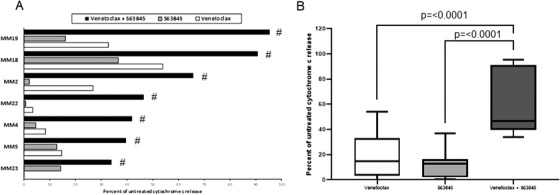
Co‐operative induction of apoptosis with the combination of venetoclax and S63845 in primary MM samples. A, Primary MM cells were treated with single agent venetoclax (1μM), S63845 (1μM) or the combination. After 4 hours, cells were fixed and processed for cytochrome c release. Hashtags indicate synergy according to the bliss independence model as described in the methods. No response to venetoclax single agent was seen in sample MM23. B, Summary of the median interquartile range (boxes) and range (error bars) of seven primary MM samples treated with venetoclax, S63845 alone or the combination (*P = <.0001)

**FIGURE 4:**
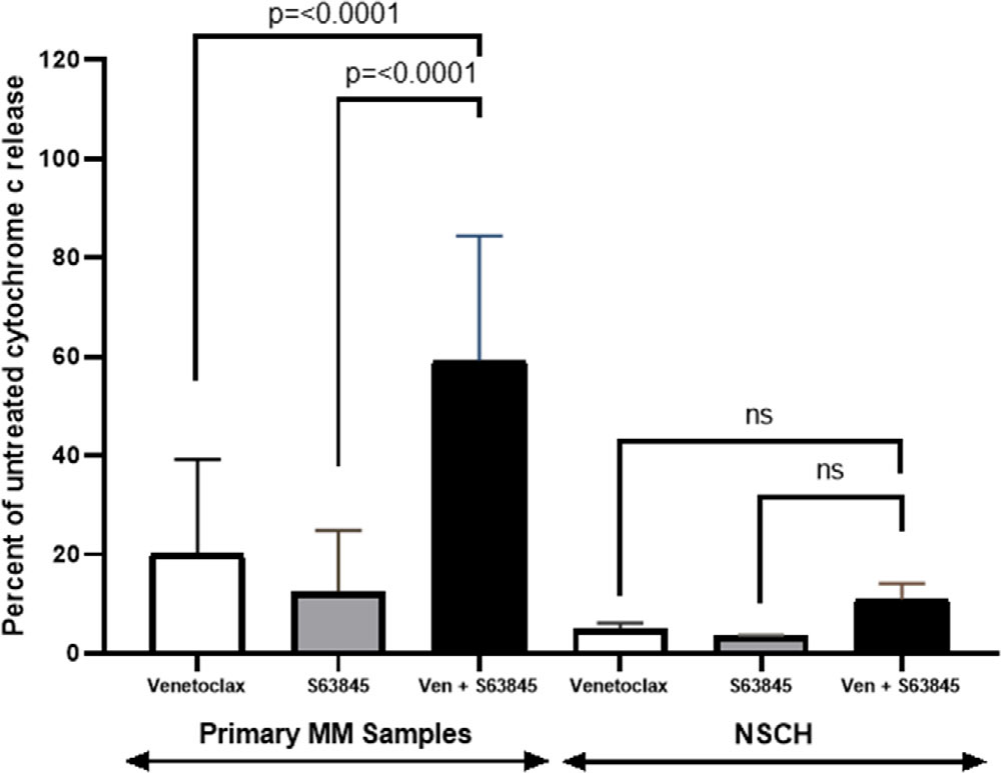
The combination of venetoclax and S63845 does not target the normal stem cell population. Seven primary MM samples and three NSCH were treated with single agent venetoclax (1μM), S63845 (1μM) or the combination. Following 4‐hours drug treatment, cells were fixed and processed for cytochrome c release (*P = <.0001)

Primary MM cells are notoriously difficult to culture ex vivo therefore confounding the use of conventional cytotoxicity assays to measure response to therapy. An assay over a short‐time frame is important as MM cells are particularly fragile in vitro, and the majority will spontaneously die in culture relatively rapidly. We found that over 96% of primary MM cells stained dual positive for Annexin‐V and propidium iodide after 24 hours under normal culture conditions (Figure S2). By employing a short‐term readout of response using intact cells we aimed to circumnavigate this issue. Bone marrow samples from seven patients with newly diagnosed MM were collected. Plasma cells were isolated and cultured for 4‐hour with venetoclax (1μM) and/or S63845 (1μM) followed by measurement of cytochrome c release (Figure 3A and B). Significant apoptotic synergy was detected in all seven primary samples tested following treatment with the drug combination for 4 hours. The apoptotic response seen in the primary samples following exposure to the drug combination for this short time was impressive and supports the use of the assay as a predictor of response to therapy. The t(4;14) translocation and gain of 1q are both considered to be high risk in MM. Of the primary samples tested one had a t(4;14) translocation whilst three carried a 1q gain suggesting that combined targeting of BCL‐2 and MCL‐1 may be beneficial in these hard to treat subgroups. To determine the specificity of the drug combination we treated NSCH with the same concentration of single agent and combination as the primary MM samples (Figure 4). Minimal apoptotic response was seen in the NSCH following treatment with the drug combination suggesting specific targeting of MM cells and limited collateral damage.

## 4 DISCUSSION

Treatment options for MM patients have increased dramatically over the last decade but assays that predict which drug an individual will best respond to are still lacking. We have previously demonstrated that primary AML cells survive poorly in vitro due to spontaneous apoptosis and show here that the same is true for primary MM cells, thus confounding the use of traditional cytotoxicity assays to determine response to therapeutics [19]. We have also previously reported two short‐term flow cytometric assays that predict long‐term chemo‐responsiveness to drugs in cell lines [17]. We investigated whether a 4‐hour cytochrome c release assay could predict long‐term response to drugs in MM cells. Targeting pro‐survival members of the BCL‐2 family is an attractive therapeutic option in MM but reliance on BCL‐2, MCL‐1 and BCL‐_XL_ for survival has been shown to be variable (Touzeau et al 2014). Venetoclax is a clinically established BCL‐2 targeting BH3‐mimetic that has FDA approval for use in CLL patients with a 17p deletion and in combination with low‐dose cytarabine or hypomethylating agents, in the treatment of AML [20, 21, 22, 23]. Pre‐clinical data showed that sensitivity to venetoclax was associated with t(11;14) and high expression of BCL‐2 in both HMCL and primary myeloma samples. Subsequent data have shown that targeting multiple BCL‐2 family members at the same time could be beneficial across all myeloma groups [12]. Clinically, venetoclax has demonstrated efficacy as targeted therapy for t(11;14) in relapsed/refractory myeloma, both as monotherapy and when combined with dexamethasone [7, 24]. However, response rates were only 40‐45% suggesting the need for additional biomarkers to predict venetoclax sensitivity. The importance of such biomarkers is highlighted by analysis of BELLINI, a phase 3 study comparing venetoclax versus placebo in combination with bortezomib and dexamethasone in relapsed myeloma. Venetoclax was found to increase response rates and progression‐free survival across all subgroups versus placebo, particularly in patients harboring t(11;14). There was a trend for increased overall survival with venetoclax in the t(11;14) subgroup. However, for non‐t(11;14) patients there was a trend towards worse overall survival if treated with venetoclax [25]. These results, along with the fact that only 15‐20% of myeloma patients will harbour a *t*(11;14), highlight the importance of reliable biomarkers to predict response to venetoclax and its combination with other therapeutics. Resistance to venetoclax monotherapy has been reported in other haematological malgnancies and is associated with pro‐apoptotic BIM, being released from BCL‐2, which is then sequestered by over‐expressed MCL‐1 or BCL‐_XL_ anti‐apoptotic proteins [26]. Given 75‐80% of MM cases rely on elevated MCL‐1 expression to evade apoptosis, dual targeting, along with BCL‐2 seems logical in this context. S63845 is a BH3‐mimetic that targets MCL‐1, and we have previously reported that it produces a synergistic apoptotic response when used in combination with venetoclax in AML cells and other groups have demonstrated this in MM cells [12, 13]. Here we confirm that the combination of venetoclax and S63845 produces a synergystic apoptotic response in MM cells. Importantly this apoptotic response could be measured in primary samples after a short 4‐hour time point using our cytochrome c release assay which was also able to predict long‐term response to the drug combination. We report here that whilst the combination of venetoclax and S63845 resulted in synergistic apoptosis in all HMCL and primary samples, the normal stem cell population remained unchallenged. The synergistic response seen in primary samples was impressive considering the short‐time frame of the assay. The authors acknowledge that the data provided proof of concept, and that expanded cohorts to determine correlation to actual clinical outcome, and safety of the drug combination would need to be determined. We have also previously reported that drugs which indirectly target MCL‐1, when used in combination with venetoclax, produce a synergistic apoptotic response [18, 27]. A worrying caveat to targeting MCL‐1 is that deletion of MCL‐1 in mouse models resulted in bone marrow failure and myocardial toxicity [8]. It is hoped that this issue can be avoided by transiently targeting MCL‐1 with a BH3‐mimetic, and importantly simultaneous targeting of BCL‐2 and MCL‐1 with BH3‐mimetics has been reported to cause minimal toxicity to normal haematopoietic progenitor cells [28]. We confirm here that S63845 does not result in loss of MCL‐1 protein as a single agent suggesting its different and potentially clinically beneficial mechanism of action. The combination of S63845 and venetoclax resulted in MCL‐1 depletion along with BCL‐2 and BCL‐_XL_ after 4 hours in accordance with the synergism seen with the drug combination in HMCL. The drug combination also resulted in cyclin D1 and D2 protein depletion following 4‐hour treatment. Translocations resulting in over‐expression of cyclin D1, t(11;14), are associated with standard risk in MM whereas those leading to over‐expression of cyclin D2 such as t(4;14) and t(14;16) are associated with higher risk disease. Of note, was that all HMCL which over‐expressed cyclin D2 were particularly sensitive to the drug combination, whilst one of the primary samples had a t(4;14) suggesting this poor prognostic group may respond well to dual BCL‐2 and MCL‐1 targeting. MM cases with t(4;14) are associated with a higher incidence of 1q gain [29]. Gain of 1q confers poor prognosis and is seen in around 30‐40% of newly diagnosed myeloma with frequency increasing with disease progression [1, 29]. Patients with more than three copies of 1q have a worse outcome, suggesting a possible dosage effect for the genes carried on 1q [30]. The chromosomal region 1q21 contains the MCL‐1 locus, and 1q gain has been reported to confer sensitivity to MCL‐1 targeting [31]. Three of our primary samples carried extra copies of 1q. Additional copies of 1q would imply elevated expression of MCL‐1 and potentially explain the sensitivity we found with the drug combination in samples with a 1q gain and the cyclin D2 expressing primary sample and HMCL.

In conclusion, we report a same‐day assay that supports previous data showing that dual BCL‐2 and MCL‐1 targeting using BH3 mimetics is synergistic in MM. This short‐term and reproducible assay can provide a quick response profile for both BCL‐2 and dual targeting and mitigate the difficulty in culturing primary myeloma cells for long periods ex vivo. Our data provide justification for an expanded cohort and experiments to determine the clinical utility of this test. The assay would need to be used in the context of a phase I clinical trial to determine its reproducibility in predicting a clinical response.

## FUNDING INFORMATION

This work was supported by grants (refs 2021 and 2262) from Nottingham University Hospitals Charities, Nottingham, UK. Further funding was obtained from the Nottinghamshire Leukaemia Appeal for Dr Grundy and Dr Seedhouse.

## CONFLICT OF INTEREST

The authors declare that there is no conflict of interest that could be perceived as prejudicing the impartiality of the research reported.

## Supporting information

Figure S1 Anti‐apoptotic protein expression following treatment with the combination of venetoclax and S63845. MCL‐1, BCL‐2, BCL‐_XL_, Cyclin D1, and Cyclin D2 protein expression in untreated MOLP‐8 cells (Lane 1) or cells treated for 4 hours with venetoclax (Lane 2), S63845 (Lane 3) or the drug combination (Lane 4). Protein expression in untreated JJN3 cells (Lane 5) or cells treated for 4 hours with venetoclax (Lane 6), S63845 (Lane 7), or the drug combination (Lane 8). Protein expression in untreated U‐266 cells (Lane 9) or cells treated for 4 hours with venetoclax (Lane 10), S63845 (Lane 11), or the drug combination (Lane 12)

Figure S2 Rapid spontaneous apoptosis in primary MM samples under normal culture conditions. Example flow cytometry plot of a primary MM sample cultured for 24 hours followed by Annexin‐V and propidium iodide positivity analysis.

## Data Availability

The data that support the findings of this study are available from the corresponding author upon reasonable request.
