# Supplementary figures and images for: A same‐day assay predicts apoptotic response to combined BCL‐2 and MCL‐1 BH3‐mimetic targeting in multiple myeloma cells
Source: EJHaem. 2020 Nov 20;2(1):40–7. doi: 10.1002/jha2.133 (PMC9175957; doi:10.1002/jha2.133)

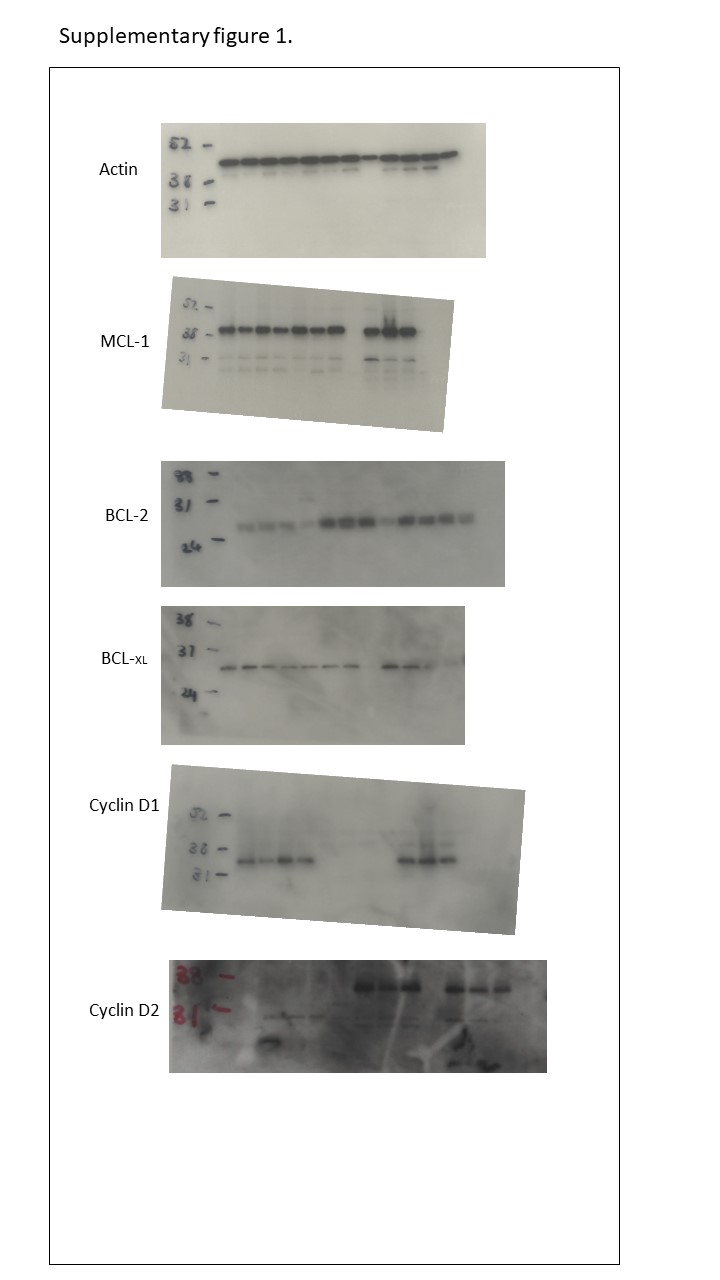

Supplement: Supplementary file 1 — Figure S1 Anti‐apoptotic protein expression following treatment with the combination of venetoclax and S63845. MCL‐1, BCL‐2, BCL‐XL, Cyclin D1, and Cyclin D2 protein expression in untreated MOLP‐8 cells (Lane 1) or cells treated for 4 hours with venetoclax (Lane 2), S63845 (Lane 3) or the drug combination (Lane 4). Protein expression in untreated JJN3 cells (Lane 5) or cells treated for 4 hours with venetoclax (Lane 6), S63845 (Lane 7), or the drug combination (Lane 8). Protein expression in untreated U‐266 cells (Lane 9) or cells treated for 4 hours with venetoclax (Lane 10), S63845 (Lane 11), or the drug combination (Lane 12) [file JHA2-2-40-s001.JPG]

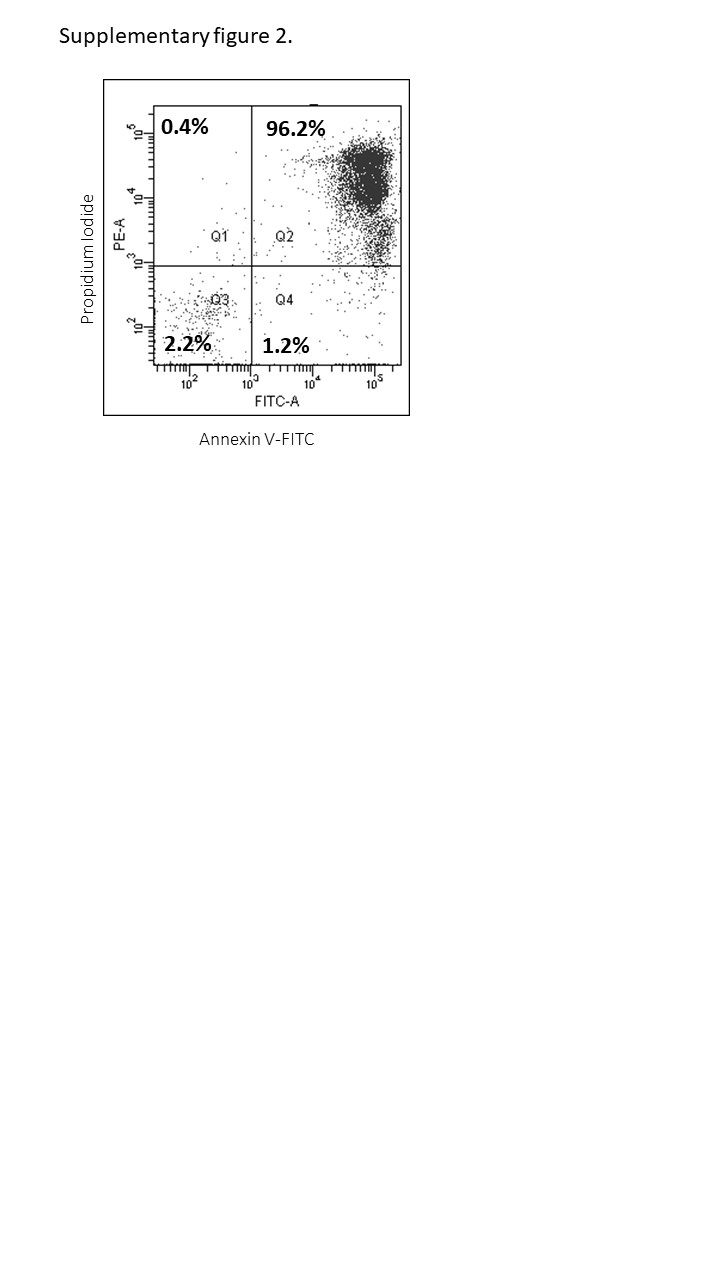

Supplement: Supplementary file 2 — Figure S2 Rapid spontaneous apoptosis in primary MM samples under normal culture conditions. Example flow cytometry plot of a primary MM sample cultured for 24 hours followed by Annexin‐V and propidium iodide positivity analysis. [file JHA2-2-40-s002.JPG]
